# Expression of a Recombinant *Lentinula edodes* Xylanase by *Pichia pastoris* and Its Effects on Ruminal Fermentation and Microbial Community in *in vitro* Incubation of Agricultural Straws

**DOI:** 10.3389/fmicb.2018.02944

**Published:** 2018-11-30

**Authors:** Lizhi Li, Mingren Qu, Chanjuan Liu, Lanjiao Xu, Ke Pan, Xiaozhen Song, Kehui OuYang, Yanjiao Li, Xianghui Zhao

**Affiliations:** Jiangxi Province Key Laboratory of Animal Nutrition/Engineering Research Center of Feed Development, Jiangxi Agricultural University, Nanchang, China

**Keywords:** recombinant *Lentinula edodes* xylanase, agricultural straws, hydrolysis, *in vitro* ruminal fermentation, microbial community

## Abstract

Agricultural straws, such as rice straw, wheat straw, and corn straw, are produced abundantly every year but not utilized efficiently in China. An experiment was conducted to determine the effects of recombinant xylanase on ruminal fermentation and microbial community structure in *in vitro* incubation of these straws. The recombinant xylanase from *Lentinula edodes* (rLeXyn11A) was produced in *Pichia pastoris*. The optimal temperature and pH for rLeXyn11A were 40°C and 4.0, respectively. The rLeXyn11A featured resistance to high temperature and showed broad temperature adaptability (>50% of the maximum activity at 20–80°C). Supplemental rLeXyn11A enhanced the hydrolysis of three agricultural straws. After *in vitro* ruminal incubation, regardless of agricultural straws, the fiber digestibility, acetate concentration, total volatile fatty acids (VFAs) production, and fermentation liquid microbial protein were increased by rLeXyn11A. Supplemental rLeXyn11A increased the ammonia-N concentration for corn straw and rice straw. High throughput sequencing and real-time PCR data showed that the effects of rLeXyn11A on ruminal microbial community depended on the fermentation substrates. With rice straw, rLeXyn11A increased the relative abundance of fibrolytic bacteria including Firmicutes, *Desulfovibrio*, Ruminococcaceae and its some genus, and *Fibrobacter succinogenes*. With wheat straw, rLeXyn11A increased the relative abundance of *Ruminococcus_1* and its three representative species *F. succinogenes, Ruminococcus flavefaciens, Ruminococcus albus*. With corn straw, the fibrolytic bacteria Firmicutes, *Christensenellaceae_R_7_group, Saccharofermentans*, and *Desulfovibrio* were increased by rLeXyn11A. This study demonstrates that rLeXyn11A could enhance *in vitro* ruminal digestion and fermentation of agricultural straws, showing the potential of rLeXyn11A for improving the utilization of agricultural straws in ruminants.

## Introduction

Some fibrous agricultural by-products, such as rice straw, wheat straw, and corn straw, are produced abundantly every year in China. These agricultural straws contain considerable quantities of cellulose and hemicellulose and has the potential to be a valuable feed source for ruminants, but their nutritive feeding values are limited by their low ruminal degradability. Though the straws could be digested by diverse ruminal microorganisms, the fiber digestibility in rumen is still less than 50% (Vallejo et al., 2016). Consequently, the majority of these agricultural straws are as wastes either left in the field for natural decay or burnt adding to environmental pollution. The xylans were the main hemicellulosic components, averaging more than 60% in the hemicelluloses of rice straw, wheat straw, and corn straw (Sun and Tomkinson, 2002; Karimi et al., 2006; Moniz et al., 2013). Therefore, some studies tried to increase ruminal xylan digestion for improving the utilization of agricultural straws in ruminants. Xylanase is the major enzyme involved in degrading the xylan polymer of plant cell wall into soluble sugars (Togtokhbayar et al., 2015). Adding xylanase to diets of ruminants is the favorite method to improve ruminal fiber degradation resulting in increased digestible energy intake, but the results have been variable and inconsistent (Beauchemin et al., 2003). An *in vivo* study showed that supplemental xylanase increased the ruminal degradability of hemicellulose or NDF for rice straw at a high dose in dairy cows (Phakachoed et al., 2012). Some *in vitro* studies also found that addition of xylanase could improve ruminal fermentation for rice straw, wheat straw, and corn stover by increasing fiber digestion, VFA production, or affecting the rumen microbial population (Mao et al., 2013; He et al., 2015; Togtokhbayar et al., 2015). However, inconsistent results were also observed in other *in vitro* or *in vivo* reports. Some studies found that xylanase did not affect or had little impact on the *in vitro* ruminal fermentation and NDF digestibility for the three straws (Eun et al., 2006; Giraldo et al., 2007; Vallejo et al., 2015). Adding xylanase did not affect the apparent digestibility of NDF and ADF, rumen fermentation parameters, and energy and N utilization in goats fed rice straw as forage (Lu et al., 2015). Beauchemin et al. (2003) pointed out that some factors responded for the inconsistent results including enzyme source, the type and dose of enzyme, composition of diet, enzyme application method and even the level of animal productivity.

White-rot fungi are the most efficient known lignocellulose degrader in nature (Zhao et al., 2015). As one of white-rot fungi, *Lentinula edodes* can use its enzymatic machineries to break down lignocellulose and improve nutritive value of low quality feeds, such as rape straw, wheat straw, rice straw, corn stover, and sugarcane bagasse, but also resulted in the great losses of cellulose and hemicellulose during the degradation, which limits its practical use (Tuyen et al., 2012, 2013; Zhao et al., 2015). However, at least it means that *L. edodes* is a good source for fibrolytic enzymes gene pool. Some studies found that significant xylanase activities were observed in *L. edodes* cultivation using agricultural straws as the growth substrates (Mata and Savoie, 1998; Tsujiyama and Ueno, 2013; Elisashvili et al., 2015). Lee et al. (2005) cloned a xylanase gene from *L. edodes* and found that the recombinant xylanase could degraded birchwood xylan into xylobiose, xylotriose, and xylotetraose (Lee et al., 2005). We hypothesize that the recombinant *L. edodes* xylanase could also enhance the hydrolysis and ruminal fermentation of agricultural straws, however, little information is available. Therefore, this study recombined, expressed and purified the *L. edodes* xylanase by *Pichia pastoris*, investigated the effects of recombinant xylanase on the hydrolysis of agricultural straws, and evaluated the effects of recombinant xylanase on *in vitro* ruminal fermentation and microbial community of agricultural straws and consequently the application possibility of *L. edodes* xylanase in the utilization of agricultural straws by ruminants.

## Materials and Methods

### Chemicals, Strains, and Conditions

*P. pastoris* strain X-33, expression vector pPICZαA, and antibiotic zeocin were purchased from Invitrogen Corporation (Invitrogen, Carlsbad, CA, United States). Restriction enzymes, pMD18-T vector, cDNA Synthesis Kit, T4 DNA ligase, PCR enzyme were obtained from TakaRa (TaKaRa Biotechnology, Dalian, China). The competent *Escherichia coli* DH5α cells and TIANprep Mini Plasmid Kit were purchased from Tiangen Biotech (Beijing) Co., Ltd (Beijing, China). Total RNA extraction kit, primers synthesis, and DNA sequencing were purchased or performed in Sangon Biotech (Shanghai) Co., Ltd (Shanghai, China). The *N*-glycosidase F (PNGase F), *O*-glycosidase, and α2-3,6,8,9 neuraminidase were purchased from NEB Biolabs (Beijing, China). Other reagents were obtained from standard commercial sources.

The *L. edodes* used for this study were obtained from local producers. The fungi was inoculated on PDA medium and incubated at 25°C until mycelia colonized most of the agar dish surface. Rice straw, wheat straw, and corn straw were ground through 0.5 mm sieves and dried at 65°C, respectively. Three straws were mixed equally. Five grams of mixed straw and 7.5 ml of water were placed in a dish and left overnight for the water to penetrate into the inner structures of straws. The wet straws were covered with cellophane paper and then sterilized in an autoclave at 121°C for 15 min. For RNA extraction, the autoclaved straws were inoculated aseptically with previously prepared mycelia and incubated at 25°C for 20 days.

### Cloning of Xylanase Gene and Construction of Expression Vector

To clone *L. edodes* xylanase gene, total RNA was extracted from mycelium grown for 20 days on the surface of the cellophane paper. cDNA was synthesized from the mycelial RNA. The primers for isolating xylanase gene were designed according to the reported gene sequences of *L. edodes* xylanase (Xyn11A; GenBank no. AF411252.1). In this study, the xylanase gene was amplified by PCR without the signal peptide sequence, using primers XynF (CCGGAATTCGTCTTTGACAACTCGAC) and XynR (GCTCTAGAAAGAGACACTGAGAATAGTACG) with restriction sites *EcoR* I and *Xba* I (underlined), respectively. Meanwhile, two base pairs (AA/TT) were introduced in the protein C-terminal domains before the *Xba* I site to avoiding a translation frameshift when the vector pPICZαA was used in this study. The PCR products were cloned into the pMD18-T vector designated pMD-Xyn and transformed into competent *Escherichia coli* DH5α cells. The pMD-Xyn plasmids were extracted, screened by PCR amplification, and sequenced by the DNA Sequencing.

The pMD-Xyn plasmid, digested with *EcoR* I and *Xba* I, was ligated into vector pPICZαA, which had been treated with the same restriction enzymes. The resulting expression vector was called pPICZαA-Xyn and transformed in to DH5α cells. Transformant was confirmed as clone by plasmid isolation, PCR amplification, restriction digestion, and DNA Sequencing for insert release. The DNA sequence data was deposited in the GenBank under accession number MH349096.

### Transformation of *P. pastoris* and Screening of Xylanase Expression Stain

Recombinant plasmid pPICZαA-Xyn was linearized by restriction digestion with *Sac* I and transformed into competent *P. pastoris* X33 by electroporation (MicroPulser; Bio-Rad, Hercules, CA, United States). The transformants were preliminarily screened on the YPD agar plates containing zeocin and incubated at 30°C for 3 days. The positive colonies were certified for integration of rLeXyn11A gene into the Pichia genome by PCR using the primers of α-factor, 3′AOX, and xylanase specific primers. For their enzyme activities, the confirmed transformants were further grown in 2 ml of BMGY medium and shaken overnight at 30°C. Meanwhile, a non-transformed *P. pastoris* X33 wild type was cultivated as a reference. The cultures were centrifuged for 5 min at 2,000 rpm. The pellets were then inoculated into 10 ml of BMMY medium in 100-ml flasks and shaken at 30°C to induce expression. During the following 1–3 days, methanol was supplemented every 24 h to the culture to a final concentration of 1.0% (v/v) for maintaining the induction. On the final day, yeast cultures were collected and centrifuged at 10,000 rpm for 5 min at 4°C. Supernatants was concentrated and changed to 0.1 M sodium acetate buffer (pH 4.5) using Amicon Ultra-4 centrifugal filter unit (Merck Millipore, Darmstadt, Germany), and assayed for the xylanase activity using oat xylan (Sigma X0627; Sigma-Aldrich) as substrates. The reaction mixture contained 100 μl supernatants, 1% (w/v) xylan, and 0.1 M sodium acetate buffer (pH 4.5). The assay was carried out at 40°C for 1 h with gentle mixing by measuring the released reducing sugars. Reducing sugar concentrations were obtained by reference to a standard curve prepared with xylose. Supernatant with the highest activity was further confirmed by sodium dodecyl sulfate polyacrylamide gel electrophoresis (SDS-PAGE) staining with Coomassie Blue. The recombinant strain with the highest expression was selected and kept for further incubation.

### Expression and Purification of Recombinant Xylanase

The recombinant strain screened was grown in 25 ml of BMGY medium and subsequent 100 ml of BMMY medium in 1 L flasks to induce expression according to aforementioned methods. The supernatant centrifuged was concentrated using Amicon Ultra-15 centrifugal filter unit. For affinity chromatography, the concentrate was diluted with a binding buffer (pH 8.0) consisting of 0.05 M sodium dihydrogen phosphate and 0.3 M sodium chloride and loaded on a 5 ml Bio-Scale Mini Nuvia IMAC Ni-Charged column (Bio-Rad, Hercules, CA, United States) connected to a low-pressure chromatographic system (Biologic LP; Bio-Rad, Hercules, CA, United States) at 1.0 ml/min. The bound xylanase was first washed with the aforementioned binding buffer containing 0.05 M imidazole (pH 8.0) and then eluted with the binding buffer containing 0.25 M imidazole (pH 8.0). The flow-through, which contained xylanase, was collected, further concentrated, and changed to water through Amicon Ultra-15 centrifugal filter unit. The concentrated protein sample was verified by Western blot and quantified with Bradford Protein Assay Kit (Sangon Biotech, Shanghai, China). SDS-PAGE was carried out to confirm the xylanase protein purity. The *N*-glycosidase F, *O*-glycosidase, and α2-3,6,8,9 neuraminidase were used to investigate the glycosylation of purified xylanase according to the manufacturer’s instructions. A zymogram technique was used to determine whether the purified xylanase had activity and isoenzymes. The zymogram technique was based on Native-PAGE and substrate gels containing remazol brilliant blue R-D-xylan (Sigma M5019; Sigma-Aldrich) and agar powder. The substrate gel was carefully overlaid with the protein gel from Native-PAGE and incubated at 40°C until observing the protein bands and clearing zone.

### Characteristics of Recombinant Xylanase

The activity of recombinant xylanase from *L. edodes* (rLeXyn11A) was determined by measuring the released reducing sugars according to the aforementioned description when the dependencies of temperature and pH for rLeXyn11A were investigated. The temperature dependency for the rLeXyn11A activity was determined by incubating reaction mixtures at 20–80°C. Determining the effects of pH on rLeXyn11A activity was performed by the reaction mixtures containing purified enzyme (8 μg), 1% (w/v) xylan, and 0.1 M sodium acetate buffer (pH 2.5–5.5) or 0.1 M sodium phosphate buffer (pH 5.5-8.0) at the optimum temperature.

### Enzymatic Hydrolysis of Agricultural Straws

Three agricultural straws including rice straw, wheat straw, and corn straw (air-dry basis) were used in this experiment. Considering the rLeXyn11A and agricultural straws would be used in the further *in vitro* rumen fermentation study (pH 6.0–7.0), the hydrolysis was performed using 8 μg purified rLeXyn11A and 20 mg straw substrates in 2 ml 0.1 M sodium phosphate buffer (pH 6.5). The mixtures were incubated in 10 ml centrifuge tubes and shaken for 24 h at 40°C as triplicates. The control groups without rLeXyn11A were incubated similarly. The reducing sugars released in samples were determined using alkaline 3,5-dinitrosalicylic acid reagent.

### *In vitro* Ruminal Fermentation

An *in vitro* study was carried out to investigate the effects of rLeXyn11A on ruminal fermentation. Rumen fluid was obtained from three ruminally fistulated beef cattle fed a diet consisting of 700 g/kg rice straw and 300 g/kg concentrates before morning feeding. The rumen liquid collected was filtered through four lays of cheesecloth and mixed (1:2 v/v) with anaerobic buffer (Cone et al., 1996). All manipulations were done under continuous flushing with CO_2_. Fermentation was conducted in 120-ml serum bottles to which 500 mg of agricultural straws and 60 ml of buffered rumen fluid were added (Cone et al., 2008). Two hundred microgram purified rLeXyn11A was added into the incubation bottles as xylanase treatments, and the same amount of water instead of xylanase were incubated similarly as controls. Bottles were closed and incubated in a shaking water bath at 39°C for 48 h. All samples were incubated in triplicate. A blank (rumen fluid without sample) was incubated in duplicate for correction of residual DM in samples. The gas produced during fermentation was recorded and expelled per 12 h. Fermentation was terminated by placing the bottles on the ice, and the residue was filtered using pre-weighted nylon bag (37 μm pore size) for the determination of IVNDFD. Samples of filtrate were determined the ruminal pH immediately. One milliliter of ruminal fluid was preserved by adding 1 ml of deproteinizing solution (100 g/L metaphosphoric acid and 0.6 g/L crotonic acid) to determine VFAs. Ten milliliter of filtrate was preserved to determine ammonia-N concentration and microbial protein synthesis. The filtrate samples were also collected and stored at -80°C for DNA extraction and microbial community determination.

### Analytical Procedures

The samples were analyzed for DM by drying at 65°C for 72 h. The NDF content in samples were analyzed according to Van Soest et al. (1991). Heat stable amylase was not used for NDF determination, and NDF was not corrected for ash content. Ammonia-N in the samples was analyzed according to Weatherburn (1967). The VFA concentrations in the filtered samples were determined by a gas chromatography (GC-2014; Shimadzu Corp., Kyoto, Japan). Crotonic acid was used as an internal standard. FLMCP synthesis was determined according to Makkar et al. (1982).

The ruminal microbiota was determined by the high-throughput sequencing at Gene Denovo Co. (Guangzhou, China). Microbial genomic DNA was extracted from fermentation liquid using the E.Z.N.A. stool DNA Kit (Omega Bio-tek, Norcross, GA, United States). The 16S rDNA V3-V4 region of the Eukaryotic ribosomal RNA gene were amplified by PCR using primers 341F (CCTACGGGNGGCWGCAG) and 806R (GGACTACHVGGGTATCTAAT) for all DNA samples. The purified PCR products were quantified by QuantiFlour^TM^ fluorimeter. Then, a composite sequencing library was generated by pooling in equimolar ratios of amplicons and sent for sequencing on an Illumina Hiseq 2500 platform. The raw Fastq data was processed and analyzed. OTUs were clustered with a 97% similarity cutoff from the clean Fastq data and chimeric sequences were identified and removed using UPARSE^1^. These OTUs were used for diversity (Shannon and Simpson), richness (Ace and Chao), and rarefaction curve analysis using Mothur. Representative sequences of OTUs were aligned to the Silva database for bacteria taxonomic assignments using QIIME software (Quast et al., 2013). PCoA was applied on the Bray-Curtis distances to generate two-dimensional plots. The raw data were deposited in the NCBI SRA under accession number SRP148540.

The 16S rDNA copy numbers of total bacteria and three fibrolytic bacterial species were determined according to Zhao et al. (2013). Briefly, the PCR primer sequences used for total bacteria were CGGCAACGAGCGCAACCC and CCATTGTAGCACGTGTGTAGCC; *Fibrobacter succinogenes*, GTTCGGAATTACTGGGCGTAAA and CGCCTGCCCCTGAACTATC (Denman and Mcsweeney, 2006); *Ruminococcus flavefaciens*, TCTGGAAACGGATGGTA and CCTTTAAGACAGGAGTTTACAA; *Ruminococcus albus*, CCCTAAAAGCAGTCTTAGTTCG and CCTCCTTGCGGTTAGAACA (Koike and Kobayashi, 2001). Quantitative real-time PCR (qPCR) was performed using a Bio-Rad CFX Connect Real-Time System (Bio-Rad Laboratories, Hercules, CA, United States), with SYBR Green chemistry and amplification program: one cycle of 95°C for 30 s, and 40 cycles of 95°C for 5 s, 55°C for 30 s for annealing and 72°C for 30 s for extension. Assays for all experimental samples were performed in triplicate. Copy numbers of each bacterial group were calculated based on their, respectively, standard curve obtained by the plasmid DNA contains cloned each targeted 16S rRNA gene fragment. The relative abundances of three fibrolytic bacterial species were obtained by calculating the ratio between them and total bacteria using gene copy numbers. The gene copy numbers of total bacteria were transformed by log_10_ before processing for analysis.

### Statistical Analyses

Statistical analysis was performed using a one-way ANOVA in IBM SPSS statistics version 20 (IBM, Chicago, IL, United States). Significance was declared at *P* ≤ 0.05. LEfSe method was used to identify the most differentially abundant taxons between groups (Segata et al., 2011) and LDA ≥ 2.5 was chosen to indicate significant difference during the analysis of high-throughput sequencing data.

## Results

### Cloning of *L. edodes* Xylanase Gene

The gene encoding xylanase from *L. edodes* without signal peptide were amplified by PCR using designed DNA primers, and the DNA sequence was determined by sequencing. The xylanase gene in the present study was found to have the high gene similarity to the reference sequence (GenBank no. AF411252.1) at 99.2% (Supplementary Figure S1), whereas the amino acid similarity between them was 99.6%, suggesting that current xylanase may possess similar enzymatic properties with that reported by Lee et al. (2005). However, the amino acid residue 182 was Ile in the reference sequence differed from the Met in the current study, which could maybe result in some differences between the xylanase from the two sequences.

### Expression of Recombinant Xylanase

**FIGURE 1:**
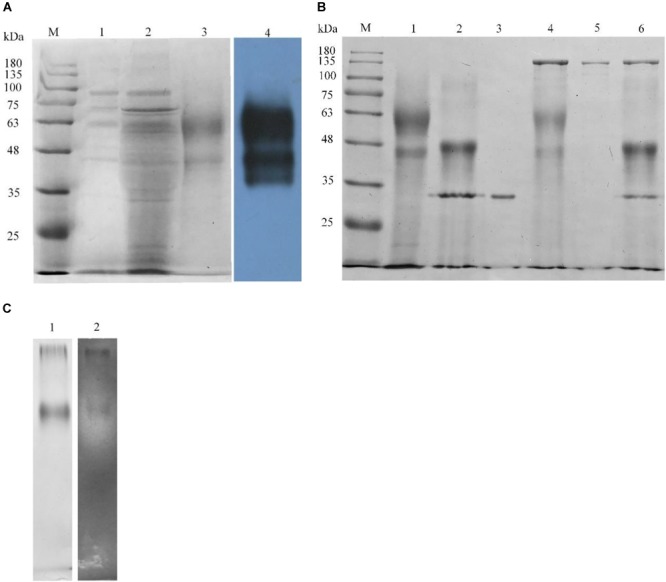
Analysis of recombinant rLeXyn11A by SDS-PAGE and Western blot **(A)**, deglycosylation **(B)**, and zymogram **(C)**. **(A)** M, protein marker; 1, concentrated culture supernatant from *P. pastoris* X33 wild type; 2, concentrated culture supernatant from rLeXyn11A transformant; 3, purified rLeXyn11A; 4, Western blot analysis of culture supernatant from rLeXyn11A transformant. **(B)** M, protein marker; 1, purified rLeXyn11A; 2, rLeXyn11A treated with *N*-glycosidase F; 3, *N*-glycosidase F; 4, rLeXyn11A treated with *O*-glycosidase and neuraminidase; 5, *O*-glycosidase and neuraminidase; 6, rLeXyn11A treated with *N*- and *O*-linked glycosidase and neuraminidase. **(C)** 1, Native-PAGE of purified rLeXyn11A; 2, zymogram of purified rLeXyn11A.

The pPICZαA-Xyn vector was constructed and transformed into competent *P. pastoris* X33 for the expression of rLeXyn11A. The rLeXyn11A expressed and purified was verified by SDS-PAGE and Western blot (Figure 1A). As shown by Figure 1A, two intense protein bands at about 59 kDa and 44 kDa were observed in the supernatant of the recombinant strain compared with the wild type, which was proved to be the rLeXyn11A protein by further Western blot analysis. The glycosylation of purified rLeXyn11A was verified using glycosidase (Figure 1B). *N*-glycosidase treatment led to a shift in migration, but *O*-glycosidase and neuraminidase (the two enzymes both are required to cleave *O*-linked disaccharides) did not cause a significant shift in migration. Concomitant treatment with *N*- and *O*-linked glycosidase, and neuraminidase also did not led to a further shift compared with *N*-glycosidase treatment. The zymogram result showed that only a protein band could degrade the xylan substrate and form a clearing zone (Figure 1C).

### Characteristics of Recombinant Xylanase

**FIGURE 2:**
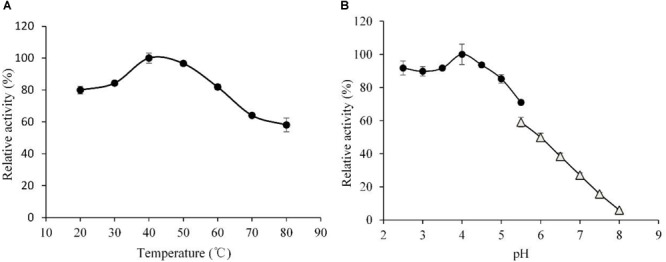
Determination of the optimal temperature **(A)** and the optimal pH **(B)** of the purified recombinant rLeXyn11A. Substrate concentration was 1% (w/v) xylan, and purified rLeXyn11A was add to a final protein loading of 0.4 μg/mg substrate in a total reaction volume of 2 ml over 1 h. **(A)** Reactions were done in 0.1 M sodium acetate buffer (pH 4.5), at 20–80°C; **(B)** reactions were done in 0.1 M sodium acetate buffer (pH 2.5–5.5) or 0.1 M sodium phosphate buffer (pH 5.5–8.0), at 40°C.

Effects of temperature and pH on hydrolytic activities of the rLeXyn11A were examined (Figure 2). The xylanase showed maximum activity at 40°C. Though the activity decreased dramatically at 80°C, it still kept the 59% of maximal activity, which suggested that the rLeXyn11A in the present study featured resistance to high temperature and showed broad temperature adaptability (>50% of the maximum activity at 20–80°C). The rLeXyn11A exhibited maximal activity at pH 4.0, but the activity decreased sharply at pH 5.5–8.0 in sodium phosphate buffer.

### Enzymatic Hydrolysis of Agricultural Straws

**FIGURE 3:**
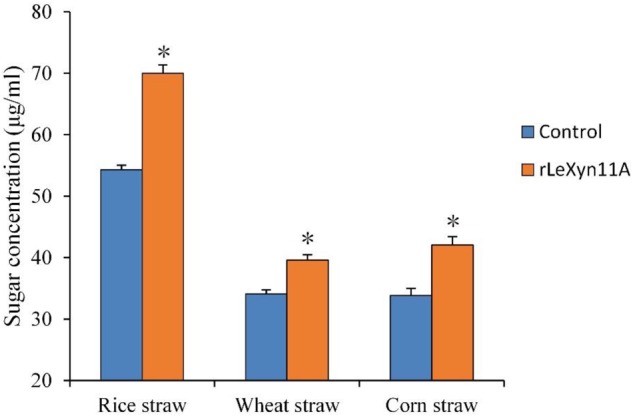
The hydrolysis activity of purified recombinant rLeXyn11A on three agricultural straws. Reactions were done in 0.1 M sodium phosphate buffer (pH 6.5), at 40°C in a total volume of 2 ml over 24 h. Three straw substrate concentration were 20 mg, respectively. Control, the straws without rLeXyn11A supplementation were shown as references; rLeXyn11A, the purified rLeXyn11A was added to a final loading of 0.4 μg/mg agricultural straws. Asterisks indicate significant differences (*P* ≤ 0.05).

Effects of rLeXyn11A on the hydrolysis of agricultural straws were investigated (Figure 3). The rLeXyn11A enhanced significantly the hydrolysis of all agricultural straws. Consequently, the reducing sugars content was increased by 28.9%, 16.2%, and 24.3% during the enzymolysis of rice straw, wheat straw, and corn straw, respectively, relative to the control group.

### Ruminal Fermentation and Microbial Community in *in vitro* Cultures of Agricultural Straws

Effects of rLeXyn11A on ruminal fermentation and microbial community in *in vitro* cultures of agricultural straws were investigated. After incubation of 48 h, regardless of agricultural straws, the ruminal pH was not affected by supplemental rLeXyn11A, averaging 6.74 (data not shown). The rLeXyn11A increased IVNDFD of corn straw, rice straw, and wheat straw by 8.8%, 3.5%, and 6.3%, respectively, compared with controls (Table 1). The acetate molar proportion was greater in rLeXyn11A-added agricultural straws than in the controls, which resulted in greater total VFA production observed in the treatments. With rice straw and wheat straw, rLeXyn11A did not affect the molar proportions of propionate and butyrate. With corn straw, however, supplemental rLeXyn11A increased the propionate molar proportion and did not affect the butyrate molar proportion relative to the control. The other VFAs were increased by rLeXyn11A in wheat straw and were not affected in corn straw and rice straw. Increases in the ammonia-N concentration were observed for corn straw and rice straw when rLeXyn11A were added. The rLeXyn11A increased FLMCP synthesis in incubations of corn straw, rice straw, and wheat straw by 11.2%, 9.4%, and 20.1%, respectively.

**Table 1:** Effects of rLeXyn11A on rumen digestion and fermentation parameters of agricultural straws in *in vitro* incubation.

Item	Rice straw		SEM	Wheat straw		SEM	Corn straw		SEM
	Control	rLeXyn11A		Control	rLeXyn11A		Control	rLeXyn11A	
IVNDFD (%)	62.10	64.29*	0.55	62.44	66.35*	1.02	56.83	61.80*	1.17
Total VFA (m*M*)	54.63	62.99*	2.10	64.70	67.65	0.82	64.57	68.16*	0.88
**Individual (mol/100 mol total VFA)**					
Acetate	60.10	70.34*	2.57	71.79	75.64*	1.05	70.94	74.73*	1.01
Propionate	14.46	15.54	0.44	16.27	16.83	0.16	16.44	17.81*	0.33
Butyrate	3.78	4.27	0.20	4.30	4.26	0.06	4.26	4.36	0.08
Other VFAs	2.31	1.84	0.12	2.52	2.60*	0.03	3.10	3.11	0.02
Ammonia-N (m*M*)	8.56	10.69*	0.49	10.95	11.71	0.26	11.96	14.03*	0.48
FLMCP (μg/ml)	296.94	324.89*	6.95	295.18	354.60*	14.30	312.02	346.89*	8.17

*SEM, standard error of the means; IVNDFD, *in vitro* NDF digestibility; Asterisks indicate significant differences (*P* ≤ 0.05); VFA, volatile fatty acids; the other VFAs including valerate, isobutyrate and isovalerate; FLMCP, fermentation liquid microbial protein.*

**FIGURE 4:**
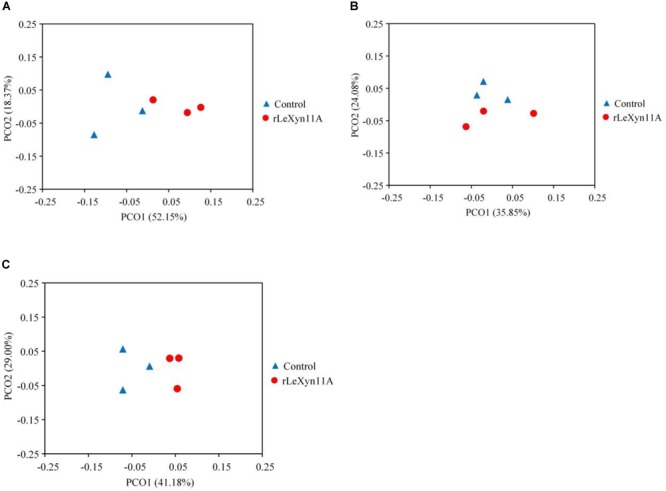
PCoA plot of samples from *in vitro* ruminal incubation of rice straw **(A)**, wheat straw **(B)**, and corn straw **(C)** based on Bray–Curtis distances. The PCoA analysis was performed using the OTUs obtained.

The high-throughput sequencing technology was used to investigate the effects of rLeXyn11A on microbial community in *in vitro* ruminal fermentation of agricultural straws. A total of 2,349,720 high-quality sequences were generated from the 18 samples. These sequences were assigned averagely to 2,401 OTUs according to the 97% sequence identity. The statistical results indicated there were no significant differences in Ace, Chao, Simpson, and Shannon indices between control and rLeXyn11A groups in the incubation of rice straw and wheat straw (Table 2). With corn straw, however, the Simpson index was greater for rLeXyn11A group than that for control group. Most rarefaction curves for each sample approached the saturation plateau, and the results of Good’s coverage showed that 99.4% of the microbial species were sampled for the microorganisms of six treatments, indicating that the sampling effort had sufficient sequence coverage for each group. PCoA based on Bray–Curtis metric was performed in order to explore dissimilarities in microbial composition between rLeXyn11A and control groups (Figure 4). The results showed that the microbiota clustered separately and axes accounted for 70.5%, 59.9%, and 70.2% of the total variation detected for rice straw, wheat straw, and corn straw, respectively, suggesting that certain key bacterial species may characterize microbiota of rLeXyn11A groups.

**Table 2:** Effects of rLeXyn11A on alpha diversity indices of rumen microbiota in *in vitro* incubation of agricultural straws.

Item	OTUs	Chao1	Ace	Coverage	Shannon	Simpson
**Rice straw**					
Control	2361	3374	3225	0.994	7.96	0.980
rLeXyn11A	2506	3425	3375	0.995	8.04	0.979
SEM	72.4	80.4	87.8	0.000	0.063	0.002
*P*	0.376	0.259	0.453	0.155	0.604	0.805
**Wheat straw**					
Control	2379	3261	3195	0.994	7.98	0.978
rLeXyn11A	2325	3180	3141	0.994	8.07	0.981
SEM	31.3	45.5	37.5	0.000	0.047	0.001
*P*	0.443	0.086	0.537	0.183	0.347	0.192
**Corn straw**					
Control	2411	3209	3175	0.994	7.93	0.977
rLeXyn11A	2428	3314	3207	0.995	8.01	0.982^∗^
SEM	30.6	43.2	50.4	0.000	0.048	0.002
*P*	0.811	0.816	0.793	0.002	0.473	0.048

*SEM, standard error of the means; asterisks indicate significant differences (*P* ≤ 0.05).*

**FIGURE 5:**
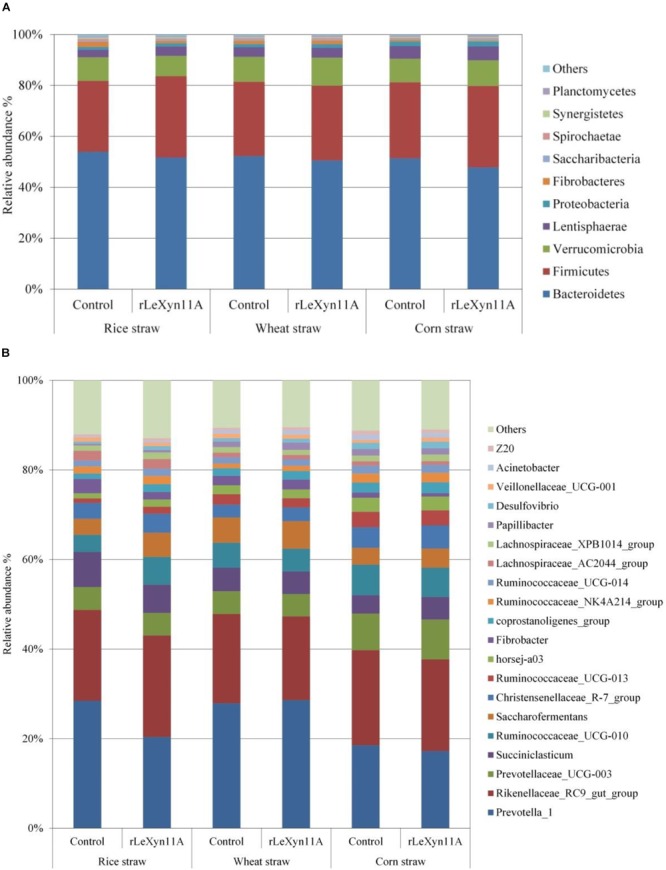
Distribution of the predominant rumen bacteria at phylum level **(A)** and genus level **(B)**. The color-coded bar plot represent the top 10 abundant taxa in the phylum level and the top 20 abundant taxa in the genus level.

**FIGURE 6:**
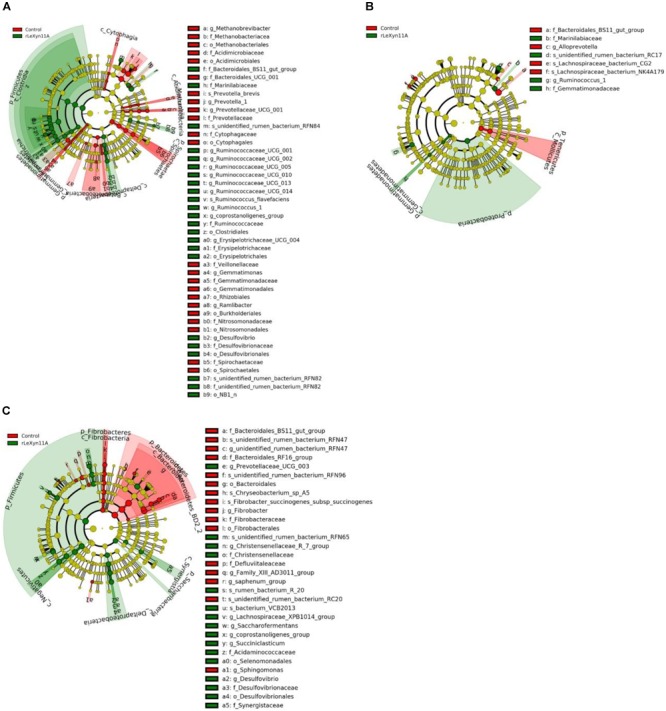
LEfSe analysis displaying the ruminal bacteria change between control group and rLeXyn11A group in *in vitro* ruminal incubation of rice straw **(A)**, wheat straw **(B)** and corn straw **(C)**. LDA ≥ 2.5 and *P* ≤ 0.05 were shown.

Based on the Silva taxonomic database and using the analysis program Uparse, the bacterial OTUs were classified and assigned to 27 phylum and 240 genus in the present study. The dominant bacterial phylum was Bacteroidetes (47.8–53.9%), followed by Firmicutes (27.9–32.0%) in all groups (Figure 5A). At the genus level, *Rikenellaceae_RC9_gut_group* (11.2–13.0%) and *Prevotella_1* (9.5–17.9%) were the dominant bacteria (Figure 5B). To identify the taxon distributions in different groups, LEfSe analysis was performed and differentially abundant taxa were found in both rLeXyn11A and control groups for three straws (Figure 6). There were 58, 13, and 42 differentially abundant taxa found for rice straw, wheat straw, and corn straw, respectively. With rice straw, 27 taxa were increased by rLeXyn11A, among which Firmicutes, *Desulfovibrio*, and some genus belonging to Ruminococcaceae such as *Ruminococcaceae_UCG_010, Ruminococcaceae_UCG_014, Ruminococcaceae_UCG_013* and so on, were found to be predominant bacteria at phylum and genus levels; 31 taxa were reduced by rLeXyn11A, among which Spirochaetae and *Prevotella_1* were the predominant bacteria. With wheat straw, seven taxa were increased by rLeXyn11A, among which phylum Proteobacteria and genus *Ruminococcus_1* were found to be predominant bacteria; Tenericutes and *Alloprevotella* were reduced by rLeXyn11A. With corn straw, 21 taxa were increased by rLeXyn11A, among which Firmicutes, Saccharibacteria, *Prevotellaceae_UCG_003, Christensenellaceae_R_7_group, Succiniclasticum, Saccharofermentans, coprostanoligenes_group*, and *Desulfovibrio* were found to be predominant bacteria; 21 taxa were reduced by rLeXyn11A, among which Bacteroidetes, Fibrobacteres, and *Fibrobacter* were the predominant bacteria.

**FIGURE 7:**
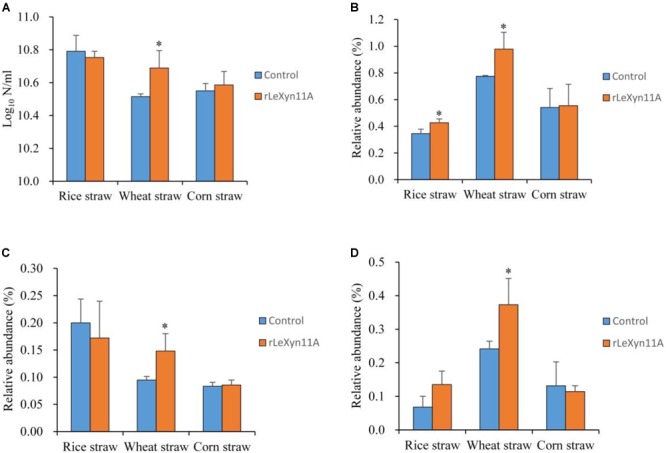
Effects of rLeXyn11A on 16S rDNA gene copy numbers of total bacteria **(A)** and relative abundances of *F. succinogenes* **(B)**, *R. flavefaciens* **(C)**, and *R. albus* **(D)** from fermentation liquid of agricultural straws in *in vitro* cultures. Log_10_ N/ml, log_10_ of 16S rDNA gene copy numbers per ml fermentation liquid; Relative abundance, 16S rDNA gene copy numbers of each predominant ruminal cellulolytic bacteria/16S rDNA gene copy numbers of total bacteria. Asterisks indicate significant differences (*P* ≤ 0.05).

Besides the high-throughput sequencing technology, the quantity of total bacteria and relative abundance of three representative fibrolytic bacteria species in the fermentation liquid was determined using real-time PCR (Figure 7). With rice straw, rLeXyn11A did not affect the 16S rDNA copy numbers of total bacteria, but significantly increased or tended to increase the relative abundance of *F. succinogenes* and *R. albus*, respectively. With wheat straw, the 16S rDNA copy numbers of total bacteria and relative abundance of three fibrolytic bacteria were all increased by supplemental rLeXyn11A. With corn straw, no differences in total bacteria and three fibrolytic bacteria were observed between control and rLeXyn11A groups.

## Discussion

*L. edodes* can use its enzymatic machineries to efficiently degrade cellulose and hemicellulose of agricultural straws, but the products are used for the growth of its spawn or sporophore, which results in the great losses of nutritional value of straws (Mata and Savoie, 1998; Tuyen et al., 2013; Zhao et al., 2015). To improve the utilization of agricultural straws, a recombinant xylanase from *L. edodes* was produced using *P. pastoris* and its effects on ruminal fermentation and microbial community was investigated in current study. Interestingly, the secreted rLeXyn11A exhibited two protein bands, of which both were apparently greater than the 27 kDa theoretical molecular mass based on the gene sequence. These maybe resulted partly from the increased glycosylation of rLeXyn11A when expressed in *P. pastoris*. Two potential *N*-glycosylation sites and 27 potential *O*-β-GlcNAc attachment sites were found in the xylanase sequence using the NetNGlyc 1.0^2^ and YinOYang 1.2 tools^3^. Thus, *P. pastoris* maybe produced rLeXyn11A as a hyperglycosylated protein. The results from glycosidase treatment confirmed our suspicion. *N*-glycosidase treatment led to a shift in migration, implying that rLeXyn11A was *N*-linked glycosylated. Concomitant treatment with *N*- and *O*-linked glycosidase, however, did not led to a further shift, suggesting that rLeXyn11A might be affected minimally by *O*-linked glycosylation. Similar to current results, the glycosylated xylanase were also expressed by *P. pastoris* in the study by He et al. (2012). Surprisingly, only a clearing zone was observed around the protein band at about 59 kDa, which meant that the protein at about 44 kDa had no xylanase activity. In addition, the protein band with small molecular weight became hard to trace after *N*-glycosidase treatment, which was very confusing and warranted further investigation.

The rLeXyn11A showed maximum activity at 40°C and featured resistance to high temperature. Current results were different from the report by Lee et al. (2005), in which the activity for recombinant *L. edodes* xylanase was maximum at 50°C and was less than 10% of maximal activity at 65°C. Different expression vector, *P. pastoris* strain, the presence or absence of native signal peptide, and the amino acid sequence of *L. edodes* xylanase used in the two studies may be responsible for the discrepant temperature dependency of rLeXyn11A (Blanchard et al., 2008; Yang et al., 2015; Chen et al., 2016). The rLeXyn11A exhibited maximal activity at pH 4.0. Similar to the result, the optimum pH for recombinant *L. edodes* xylanase was 4.5 in the study by Lee et al. (2005).

The rLeXyn11A enhanced significantly the hydrolysis of all agricultural straws in the current study. Considering few references about the direct relation between *L. edodes* xylanase and agricultural straws, this paper compared our results with those obtained using xylanase from other microorganisms. Similar to our results, previous studies also found that xylanase from some bacteria or fungus exhibited high hydrolytic efficiency toward agricultural straws (Zilliox and Debeire, 1998; Zhang et al., 2011; Dutta et al., 2014). In addition, the dramatical ability of rLeXyn11A to hydrolyze agricultural straws may help to explain why significant xylanase activities were detected in *L. edodes* cultivation using agricultural straws (Tsujiyama and Ueno, 2013; Elisashvili et al., 2015).

Current results showed that rLeXyn11A supplementation increased the IVNDFD and production of acetate and total VFA during the incubations of agricultural straws. These results were partially consistent with the previous reports (Mao et al., 2013; He et al., 2015; Togtokhbayar et al., 2015), in which exogenous xylanase improved the *in vitro* fermentation parameters of three straws including VFA and/or NDF digestibility. The increased IVNDFD might be due to the accelerated enzymatic hydrolysis of cellulose and/or hemicellulose in the three straws caused by rLeXyn11A. Corresponding with the deduction, the acetate molar proportion, total VFA concentration, and fibrolytic bacteria were significantly increased by supplemental rLeXyn11A regardless of straws. In the rumen, VFAs are produced as end-products of microbial fermentation and dietary carbohydrates, i.e., cellulose, hemicellulose, pectin, starch and soluble sugars, are the main fermentation substrates (Dijkstra, 1994). The relative concentrations of the individual VFAs, commonly referred to as the fermentation pattern, is determined by the composition of the microbial population, which in turn is largely determined by the type of dietary carbohydrate (France and Dijkstra, 2005). Fermentation of structural carbohydrates (i.e., cellulose and hemicellulose), compared to fermentation of starch, encourages the growth of acetate producing bacterial species and consequently yielded high proportion or amount of acetate (Dijkstra, 1994). The relative abundance of phylum Firmicutes and genus *Desulfovibrio* were increased by rLeXyn11A for rice straw and corn straw. It has been well-known that Firmicutes is a phylum with fibrolytic metabolic pattern, which mainly digest cellulose, hemicellulose, and some simple sugars widely existing dietary forage resulting in increased acetate concentration or proportion (Sandri et al., 2014; Klang et al., 2015). *Desulfovibrio* belongs to sulfate-reducing bacteria and could metabolize lactate and pyruvate into acetate and CO_2_ when the latter serves as an electron donor for sulfate reduction (Voordouw, 1995). Therefore, it was not surprising that the increased acetate molar proportion associated with increased relative abundance of genus *Desulfovibrio*. Besides a few common characteristics, more differences in the microbial community were observed among three straws, suggesting that the effects of rLeXyn11A on ruminal microbial structure depended on the fermentation substrates. With rice straw, the family Ruminococcaceae and its genus, i.e., *Ruminococcaceae_UCG_010, Ruminococcaceae_UCG_013, coprostanoligenes_group, Ruminococcaceae_UCG_005, Ruminococcaceae_UCG_002, Ruminococcaceae_UCG_014, Rumino coccaceae_UCG_001*, and *Ruminococcus_1*, were significantly increased by rLeXyn11A in the present study. The family Ruminococcaceae belongs to the phylum Firmicutes. Though the metabolic characters of members from Ruminococcaceae were variable, most of its genus are acetate producing bacteria and could ferment carbohydrates to acetate as the major products (Rainey, 2009a). With wheat straw, rLeXyn11A increased the relative abundance of phylum Proteobacteria and genus *Ruminococcus_1*. Proteobacteria constitutes a large group of bacteria comprising various bacterial taxa capable of catabolizing a wide range of feedstuff components (Thoetkiattikul et al., 2013). Previous results suggested that the relation between Proteobacteria and dietary fiber were controversial. The Proteobacteria abundance was reduced in some studies (Thoetkiattikul et al., 2013; Yang et al., 2013; Nobel et al., 2017), but was increased in others with supplemental high-fiber diets (Zhi et al., 2013; Zhu et al., 2015). Niu et al. (2015) found that Proteobacteria were positively correlated with apparent fiber digestibility in pigs. Current results were consistent with latter reports. For *Ruminococcus_1, R. flavefaciens, F. succinogenes*, and *R. albus* were its important members and contribute significantly to fiber metabolism (Satoshi and Yasuo, 2009). Previous *in vitro* studies showed that proliferation of the three fibrolytic bacteria was often accompanied by the increase in fiber digestion and acetate production (Sung et al., 2007; Wang et al., 2009). The fiber digestibility, acetate molar proportion, all or part of the three representative fibrolytic bacteria and consequently *Ruminococcus_1* were increased by rLeXyn11A in the incubation of wheat straw and rice straw, which were in agreement with these reports. With corn straw, besides Firmicutes and *Desulfovibrio*, rLeXyn11A also increased the relative abundance of genus *Christensenellaceae_R_7_group, Succiniclasticum*, and *Saccharofermentans*. The genus *Christensenellaceae_R_7_group* belongs to the family Christensenellaceae. Christensenellaceae could utilize various sugars and produce acetic and butyric acids as major fermentation end products (Morotomi et al., 2012). Similar to current results, abundance of Christensenellaceae was linearly increased associated with increased dietary insoluble fiber in the report by Jenkins et al. (2015). The genus *Succiniclasticum* belonging to the family Acidaminococcaceae could convert succinate to propionate but could not ferment other carbohydrates (Rainey, 2009b; Marchandin et al., 2010). Therefore, the increased *Succiniclasticum* may be relative to the increased propionate molar proportion by rLeXyn11A for corn straw. *Saccharofermentans* is known to play an important role in fiber degradation and produces acetate as main end-products (Liu et al., 2016; Zhang et al., 2017). Zhang et al. (2017) found that *Saccharofermentans* was linearly increased with increased dietary forage content and was positively associated with ruminal acetate concentration, which was consistent with the current study. Besides the increased taxa, rLeXyn11A also reduced the proportion of some taxa such as Bacteroidetes, Fibrobacteres, Spirochaetae, and so on. The microbial community was dominated by Bacteroidetes and Firmicutes regardless of groups in the current study. This is consistent with the study by De Oliveira et al. (2013), in which Bacteroidetes and Firmicutes are found predominantly in the rumen from steer. In the present study, the reduction of Bacteroidetes and other taxa may be due to the competition of faster growing cellulolytic bacteria belonging to Firmicutes in obtaining nutrients or energy (Witten and Richardson, 2003; Theodorou et al., 2005).

Supplemental rLeXyn11A increased the production of ammonia-N for rice straw and corn straw in the present study. The production of ammonia-N during fermentation depends on the extent of dietary nitrogen degradation and nitrogen uptake by ruminal bacteria. The higher production of ammonia-N in the rLeXyn11A treatments may have related to higher dietary nitrogen degradation compared with control treatments. The protein or amino-acid degrading bacteria Erysipelotrichaceae (Hugenholtz et al., 2018) and Synergistaceae (Jumas-Bilak et al., 2009) were significantly increased by rLeXyn11A for rice straw and corn straw, respectively, supporting the deduction. The optimal concentrations of ruminal ammonia-N for microbial growth are controversial, but 5 mg/dl of ammonia-N maximized microbial protein synthesis *in vitro* in previous research (Satter and Slyter, 1974). The concentrations of ammonia-N all exceeded 5 mg/dl in both rLeXyn11A and control treatments, which suggests that microbial growth may not be limited by available ammonia-N in the present study. Increased microbial protein production with supplemental rLeXyn11A may relate to the synchrony between protein and carbohydrate digestion during fermentation. Microbial protein synthesis depends largely on the available amount and fermentation rate of carbohydrates and N in the rumen (National Research Council [NRC], 2001). With enough available ammonia-N, increased NDF digestion by rLeXyn11A may stimulated the growth of more ruminal bacteria. Similarly, higher microbial protein yield due to an increase in the fermentable DM was also observed in the study by Sommart et al. (2000).

## Conclusion

In conclusion, rLeXyn11A was easily produced with the yeast *P. pastoris* and purified by affinity chromatography. The rLeXyn11A showed maximum activity at 40°C and featured resistance to high temperature. The rLeXyn11A could improve the hydrolysis and *in vitro* ruminal fermentation of agricultural straws diets by increasing the digestion of fiber, changing the structure of microbial community, stimulating the growth of fibrolytic bacteria and production of VFA, and enhancing microbial protein synthesis. The rLeXyn11A could be a promising feed additive for improving the utilization of agricultural straws in ruminants. However, it is worth noting that some limitations also exist in the present study. The positive control was lacking due to the unavailability of other *L. edodes* xylanases and consequently the new xylanase genes of *L. edodes* need to be further isolated and identified. In addition, *in vivo* rumen environment is quite complicated, and the present results obtained from *in vitro* ruminal fermentation should be confirmed using animal system before applying them to production. In order to achieve this purpose, the large scale bioreactor should be used in place of shaking flask to produce enormous amount of rLeXyn11A.

## Ethics Statement

The present study was approved by the Animal Care and Use Committee of Jiangxi Agricultural University.

## Author Contributions

XZ and LL designed the study. LL and CL conducted the study and collected the data. LX, KP, XS, KO, and YL analyzed the data. MQ helped with the manuscript writing. LL and XZ wrote the paper. XZ had primary responsibility for the final content. All authors have read and approved the final manuscript.

## Conflict of Interest Statement

The authors declare that the research was conducted in the absence of any commercial or financial relationships that could be construed as a potential conflict of interest.

## Footnotes

1 http://drive5.com/uparse/

2 http://www.cbs.dtu.dk/services/NetNGlyc/

3 http://www.cbs.dtu.dk/services/YinOYang/

## Abbreviations

ADF: acid detergent fiber
DM: dry matter
FLMCP: fermentation liquid microbial protein
IVNDFD: *in vitro* NDF digestibility
LDA: linear discriminant analysis
LEfSe: linear discriminant analysis effect size
NDF: neutral detergent fiber
OTUs: operational taxonomic units
PCoA: principal coordinate analysis
PDA: potato dextrose agar
rDNA: ribosomal deoxyribonucleic acid
SEM: standard error of the means
VFAs: volatile fatty acids

## References

[B1] Beauchemin K. A. Colombatto D. C. Morgavi D. P. Yang W. Z. (2003). Use of exogenous fibrolytic enzymes to improve feed utilization by ruminants. *J. Anim. Sci.* 81(14 Suppl. 2) E37–E47.

[B2] Blanchard V. Gadkari R. A. George A. V. E. Roy S. Gerwig G. J. Leeflang B. R. (2008). High-level expression of biologically active glycoprotein hormones in *Pichia pastoris* strains-selection of strain GS115, and not X-33, for the production of biologically active N-glycosylated 15 N-labeled phCG. *Glycoconj. J.* 25 245–257. 10.1007/s10719-007-9082-8 18274893 PMC2668595

[B3] Chen X. Hu Y. H. Chen W. D. Li W. D. Huang Z. C. Li Y. (2016). Comparison of inducible versus constitutive expression of plectasin on yields and antimicrobial activities in *Pichia pastoris*. *Protein Expr. Purif.* 118 70–76. 10.1016/j.pep.2015.10.010 26500192

[B4] Cone J. W. Gelder A. H. V. Schooten H. A. V. Groten J. A. M. (2008). Effects of forage maize type and maturity stage on in vitro rumen fermentation characteristics. *NJAS Wageningen J. Life Sci.* 55 139–154. 10.1016/S1573-5214(08)80033-4

[B5] Cone J. W. Gelder A. H. V. Visscher G. J. W. Oudshoorn L. (1996). Influence of rumen fluid and substrate concentration on fermentation kinetics measured with a fully automated time related gas production apparatus. *Anim. Feed Sci. Technol.* 61 113–128. 10.1016/0377-8401(96)00950-9

[B6] De Oliveira M. N. Jewell K. A. Freitas F. S. Benjamin L. A. Tótola M. R. Borges A. C. (2013). Characterizing the microbiota across the gastrointestinal tract of a Brazilian Nelore steer. *Vet. Microbiol.* 164 307–314. 10.1016/j.vetmic.2013.02.013 23490556

[B7] Denman S. E. Mcsweeney C. S. (2006). Development of a real-time PCR assay for monitoring anaerobic fungal and cellulolytic bacterial populations within the rumen. *FEMS Microbiol. Ecol.* 58 572–582. 10.1111/j.1574-6941.2006.00190.x 17117998

[B8] Dijkstra J. (1994). Production and absorption of volatile fatty acids in the rumen. *Livest. Prod. Sci.* 39 61–69. 10.1016/0301-6226(94)90154-6

[B9] Dutta N. Mukhopadhyay A. Dasgupta A. K. Chakrabarti K. (2014). Improved production of reducing sugars from rice husk and rice straw using bacterial cellulase and xylanase activated with hydroxyapatite nanoparticles. *Bioresour. Technol.* 153 269–277. 10.1016/j.biortech.2013.12.016 24370926

[B10] Elisashvili V. Kachlishvili E. Asatiani M. D. (2015). Shiitake medicinal mushroom, lentinus edodes (higher basidiomycetes) productivity and lignocellulolytic enzyme profiles during wheat straw and tree leaf bioconversion. *Int. J. Med. Mushrooms* 17 77–86. 10.1615/IntJMedMushrooms.v17.i1.80 25746408

[B11] Eun J. S. Hong S. H. Beauchemin K. A. Bauer M. W. (2006). Exogenous enzymes added to untreated or ammoniated rice straw: effects on in vitro fermentation characteristics and degradability. *Anim. Feed Sci. Technol.* 131 87–102. 10.1016/j.anifeedsci.2006.01.026

[B12] France J. Dijkstra J. (2005). “Volatile fatty acid production,” in *Quantitative Aspects of Ruminant Digestion and Metabolism* eds Dijkstra J. Forbes J. M. France J. (Wallingford: CABI Publishing/CAB International).

[B13] Giraldo L. A. Carro M. D. Ranilla M. J. Tejido M. L. Mohamed A. H. Priolo A. (2007). In vitro ruminal fermentation of low-quality forages as influenced by the treatment with exogenous fibrolytic enzymes. *Opt. Méditerr. Sér. A Sémin. Méditerr.* 74 263–267.

[B14] He Z. Huang Y. Qin Y. Liu Z. Mo D. Cong P. (2012). Comparison of alpha-factor preprosequence and a classical mammalian signal peptide for secretion of recombinant xylanase xynB from yeast *Pichia pastoris*. *J. Microbiol. Biotechnol.* 22 479–483. 10.4014/jmb.1109.09031 22534294

[B15] He Z. X. Yang L. Y. Yang W. Z. Beauchemin K. A. Tang S. X. Huang J. Y. (2015). Efficacy of exogenous xylanases for improving in vitro fermentation of forages. *J. Agric. Sci.* 153 538–553. 10.1017/S0021859614000860

[B16] Hugenholtz F. Davids M. Schwarz J. Müller M. Tomé D. Schaap P. (2018). Metatranscriptome analysis of the microbial fermentation of dietary milk proteins in the murine gut. *PLoS One* 13:e0194066. 10.1371/journal.pone.0194066 29664912 PMC5903625

[B17] Jenkins S. N. Waite I. S. Mansfield J. Kim J. C. Pluske J. R. (2015). Relationships between diets different in fibre type and content with growth, *Escherichia coli* shedding, and faecal microbial diversity after weaning. *Anim. Prod. Sci.* 55:1451 10.1071/ANv55n12Ab125

[B18] Jumas-Bilak E. Roudière L. Marchandin H. (2009). Description of ‘Synergistetes’ phyl. nov. and emended description of the phylum ‘Deferribacteres’ and of the family Syntrophomonadaceae, phylum ‘Firmicutes’. *Int. J. Syst. Evol. Microbiol.* 59 1028–1035. 10.1099/ijs.0.006718-0 19406787

[B19] Karimi K. Kheradmandinia S. Taherzadeh M. J. (2006). Conversion of rice straw to sugars by dilute-acid hydrolysis. *Biomass Bioenergy* 30 247–253. 10.1016/j.biombioe.2005.11.015

[B20] Klang J. Theuerl S. Szewzyk U. Huth M. Tölle R. Klocke M. (2015). Dynamic variation of the microbial community structure during the long-time mono-fermentation of maize and sugar beet silage. *Microb. Biotechnol.* 8 764–775. 10.1111/1751-7915.12263 25712194 PMC4554465

[B21] Koike S. Kobayashi Y. (2001). Development and use of competitive PCR assays for the rumen cellulolytic bacteria: *Fibrobacter succinogenes, Ruminococcus albus*, and *Ruminococcus flavefaciens*. *FEMS Microbiol. Lett.* 204 361–366. 10.1111/j.1574-6968.2001.tb10911.x 11731149

[B22] Lee C. C. Wong D. W. Robertson G. H. (2005). Cloning and characterization of the xyn11A gene from *Lentinula edodes*. *Protein J.* 24 21–26. 10.1007/s10930-004-0602-0 15756814

[B23] Liu J. H. Zhang M. L. Zhang R. Y. Zhu W. Y. Mao S. Y. (2016). Comparative studies of the composition of bacterial microbiota associated with the ruminal content, ruminal epithelium and in the faeces of lactating dairy cows. *Microb. Biotechnol.* 9 257–268. 10.1111/1751-7915.12345 26833450 PMC4767291

[B24] Lu Q. Jiao J. Tang S. He Z. Zhou C. Han X. (2015). Effects of dietary cellulase and xylanase addition on digestion, rumen fermentation and methane emission in growing goats. *Arch. Anim. Nutr.* 69 251–266. 10.1080/1745039X.2015.1039760 25963843

[B25] Makkar H. P. Sharma O. P. Dawra R. K. Negi S. S. (1982). Simple determination of microbial protein in rumen liquor. *J. Dairy Sci.* 65 2170–2173. 10.3168/jds.S0022-0302(82)82477-6 7153399

[B26] Mao H. L. Wu C. H. Wang J. K. Liu J. X. (2013). Synergistic effect of cellulase and xylanase on in vitro rumen fermentation and microbial population with rice straw as substrate. *Anim. Nutr. Feed Technol.* 13 477–487.

[B27] Marchandin H. Teyssier C. Campos J. Jeanpierre H. Roger F. Gay B. (2010). *Negativicoccus succinicivorans* gen. nov., sp. nov., isolated from human clinical samples, emended description of the family Veillonellaceae and description of Negativicutes classis nov., Selenomonadales ord. nov. and Acidaminococcaceae fam. nov. in the bac. *Int. J. Syst. Evol. Microbiol.* 60 1271–1279. 10.1099/ijs.0.013102-0 19667386

[B28] Mata G. Savoie J. M. (1998). Extracellular enzyme activities in six *Lentinula edodes* strains during cultivation in wheat straw. *World J. Microbiol. Biotechnol.* 14 513–519. 10.1023/A:1008886521091

[B29] Moniz P. Pereira H. Quilhó T. Carvalheiro F. (2013). Characterisation and hydrothermal processing of corn straw towards the selective fractionation of hemicelluloses. *Ind. Crops Prod.* 50 145–153. 10.1016/j.indcrop.2013.06.037

[B30] Morotomi M. Nagai F. Watanabe Y. (2012). Description of *Christensenella minuta* gen. nov., sp. nov., isolated from human faeces, which forms a distinct branch in the order Clostridiales, and proposal of Christensenellaceae fam. nov. *Int. J. Syst. Evol. Microbiol.* 62 144–149. 10.1099/ijs.0.026989-0 21357455

[B31] National Research Council [NRC] (2001). *Nutrient Requirements of Dairy Cattle* 7th Edn. Washington, DC: The National Academies Press.

[B32] Niu Q. Li P. Hao S. Zhang Y. Kim S. W. Li H. (2015). Dynamic distribution of the gut microbiota and the relationship with apparent crude fiber digestibility and growth stages in pigs. *Sci. Rep.* 5:9938. 10.1038/srep09938 25898122 PMC4404679

[B33] Nobel Y. R. Snider E. J. Compres G. Freedberg D. E. Toussaint N. C. Abrams J. A. (2017). Dietary fiber intake is associated with a significantly altered human esophageal microbiome. *Gastroenterology* 152:S632 10.1016/S0016-5085(17)32241-2 PMC6200756 30356041

[B34] Phakachoed N. Lounglawan P. Suksombat W. (2012). Effects of xylanase supplementation on ruminal digestibility in fistulated non-lactating dairy cows fed rice straw. *Livest. Sci.* 149 104–108. 10.1016/j.livsci.2012.07.002

[B35] Quast C. Pruesse E. Yilmaz P. Gerken J. Schweer T. Yarza P. (2013). The SILVA ribosomal RNA gene database project: improved data processing and web-based tools. *Nucleic Acids Res.* 41 590–596. 10.1093/nar/gks1219 23193283 PMC3531112

[B36] Rainey F. A. (2009a). “Family VIII. Ruminococcaceae fam. Nov,” in *Bergey’s Manual of Systematic Bacteriology* Vol. 3 eds De Vos P. Garrity G. M. Jones D. Krieg N. R. Ludwig W. Rainey F. A. Schleifer K. H. Whitman W. B. (Berlin: Springer).

[B37] Rainey F. A. (2009b). “Genus XXIII. Succiniclasticum van Gylswyk 1995, 298VP,” in *Bergey’s Manual of Systematic Bacteriology* Vol. 3 eds De Vos P. Garrity G. M. Jones D. Krieg N. R. Ludwig W. Rainey F. A. Schleifer K. H. Whitman W. B. (Berlin: Springer).

[B38] Sandri M. Manfrin C. Pallavicini A. Stefanon B. (2014). Microbial biodiversity of the liquid fraction of rumen content from lactating cows. *Animal* 8 572–579. 10.1017/S1751731114000056 24524278

[B39] Satoshi K. Yasuo K. (2009). Fibrolytic rumen bacteria: their ecology and functions. *Asian Australas. J. Anim. Sci.* 40 1141–1147.

[B40] Satter L. D. Slyter L. L. (1974). Effect of ammonia concentration on rumen microbial protein production in vitro. *Br. J. Nutr.* 32 199–208. 10.1079/BJN19740073 4472574

[B41] Segata N. Izard J. Waldron L. Gevers D. Miropolsky L. Garrett W. S. (2011). Metagenomic biomarker discovery and explanation. *Genome Biol.* 12:R60. 10.1186/gb-2011-12-6-r60 21702898 PMC3218848

[B42] Sommart K. Parker D. S. Rowlinson P. Wanapat M. (2000). Fermentation characteristics and microbial protein synthesis in an in vitro system using cassava, rice straw and dried ruzi grass as substrates. *Asian Australas. J. Anim. Sci.* 13 1084–1093. 10.5713/ajas.2000.1084

[B43] Sun R. C. Tomkinson J. (2002). Characterization of hemicelluloses obtained by classical and ultrasonically assisted extractions from wheat straw. *Carbohydr. Polym.* 50 263–271. 10.1016/S0144-8617(02)00037-1

[B44] Sung H. G. Kobayashi Y. Chang J. Ha A. Hwang I. H. Ha J. K. (2007). Low ruminal pH reduces dietary fiber digestion via reduced microbial attachment. *Asian Australas. J. Anim. Sci.* 20 200–207. 10.5713/ajas.2007.200

[B45] Theodorou M. K. France J. Forbes J. M. (2005). “Rumen microorganisms and their interactions,” in *Quantitative Aspects of Ruminant Digestion & Metabolism* eds Dijkstra J. Forbes J. M. France J. (Wallingford: CABI Pub.) 145–163. 10.1079/9780851998145.0207

[B46] Thoetkiattikul H. Mhuantong W. Laothanachareon T. Tangphatsornruang S. Pattarajinda V. Eurwilaichitr L. (2013). Comparative analysis of microbial profiles in cow rumen fed with different dietary fiber by tagged 16S rRNA gene pyrosequencing. *Curr. Microbiol.* 67 130–137. 10.1007/s00284-013-0336-3 23471692

[B47] Togtokhbayar N. Cerrillo M. A. Rodríguez G. B. Elghandour M. M. M. Y. Salem A. Z. M. Urankhaich C. (2015). Effect of exogenous xylanase on rumen in vitro gas production and degradability of wheat straw. *Anim. Sci. J.* 86 765–771. 10.1111/asj.12364 25923062

[B48] Tsujiyama S. Ueno H. (2013). Performance wood-rotting fungi-based enzymes on enzymic saccharification of rice straw. *J. Sci. Food Agric.* 93 2841–2848. 10.1002/jsfa.6118 23450755

[B49] Tuyen D. V. Phuong H. N. Cone J. W. Baars J. J. P. Sonnenberg A. S. M. Hendriks W. H. (2013). Effect of fungal treatments of fibrous agricultural by-products on chemical composition and in vitro rumen fermentation and methane production. *Bioresour. Technol.* 129 256–263. 10.1016/j.biortech.2012.10.128 23261998

[B50] Tuyen V. D. Cone J. W. Baars J. J. P. Sonnenberg A. S. M. Hendriks W. H. (2012). Fungal strain and incubation period affect chemical composition and nutrient availability of wheat straw for rumen fermentation. *Bioresour. Technol.* 111 336–342. 10.1016/j.biortech.2012.02.001 22377477

[B51] Vallejo L. H. Salem A. Z. M. Camacho L. M. Kholif A. M. Mariezcurrena M. D. Cipriano M. (2016). Effects of xylanase supplementation on feed intake, digestibility and ruminal fermentation in Rambouillet sheep. *J. Agric. Sci.* 154 1110–1117. 10.1017/S0021859616000216

[B52] Vallejo L. H. Salem A. Z. M. Kholif A. E. Elghangour M. M. Y. Fajardo R. C. Bastida A. Z. (2015). Influence of cellulase or xylanase on the in vitro rumen gas production and fermentation of corn stover. *Indian J. Anim. Sci.* 68 70–74.

[B53] Van Soest P. J. Robertson J. B. Lewis B. A. (1991). Methods for dietary fiber, neutral detergent fiber, and nonstarch polysaccharides in relation to animal nutrition. *J. Dairy Sci.* 74 3583–3597. 10.3168/jds.S0022-0302(91)78551-2 1660498

[B54] Voordouw G. (1995). The genus desulfovibrio: the centennial. *Appl. Environ. Microbiol.* 61 2813–2819. 16535089 10.1128/aem.61.8.2813-2819.1995 PMC1388543

[B55] Wang Y. Trevorw A. Tima M. A. (2009). In vitro effects of phlorotannins from *Ascophyllum nodosum* (brown seaweed) on rumen bacterial populations and fermentation. *J. Sci. Food Agric.* 89 2252–2260. 10.1002/jsfa.3717

[B56] Weatherburn M. W. (1967). Phenol-hypochlorite reaction for determination of ammonia. *Anal. Chem.* 39 971–974. 10.1021/ac60252a045

[B57] Witten G. Q. Richardson F. D. (2003). Competition of three aggregated microbial species for four substrates in the rumen. *Ecol. Model.* 164 121–135. 10.1016/S0304-3800(02)00383-6

[B58] Yang J. Martínez I. Walter J. Keshavarzian A. Rose D. J. (2013). In vitro characterization of the impact of selected dietary fibers on fecal microbiota composition and short chain fatty acid production. *Anaerobe* 23 74–81. 10.1016/j.anaerobe.2013.06.012 23831725

[B59] Yang M. Teymorian S. Olivares P. Ppn M. (2015). Extracellular expression of alkaline phytase in *Pichia pastoris*: influence of signal peptides, promoters and growth medium. *Biotechnol. Rep.* 6 112–118. 10.1016/j.btre.2015.03.005 28626704 PMC5466264

[B60] Zhang J. Shi H. Wang Y. Li S. Cao Z. Ji S. (2017). Effect of dietary forage to concentrate ratios on dynamic profile changes and interactions of ruminal microbiota and metabolites in holstein heifers. *Front. Microbiol.* 8:2206. 10.3389/fmicb.2017.02206 29170660 PMC5684179

[B61] Zhang J. Siika-Aho M. Puranen T. Tang M. Tenkanen M. Viikari L. (2011). Thermostable recombinant xylanases from *Nonomuraea flexuosa* and *Thermoascus aurantiacus* show distinct properties in the hydrolysis of xylans and pretreated wheat straw. *Biotechnol. Biofuels* 4:12. 10.1186/1754-6834-4-12 21592333 PMC3114720

[B62] Zhao X. Gong J. Zhou S. Ouyang K. Song X. Fu C. (2015). Effect of fungal treatments of rape straw on chemical composition and *in vitro* rumen fermentation characteristics. *Bioresources* 10 622–637.

[B63] Zhao X. H. Liu C. J. Liu Y. Li C. Y. Yao J. H. (2013). Effects of replacing dietary starch with neutral detergent-soluble fibre on ruminal fermentation, microbial synthesis and populations of ruminal cellulolytic bacteria using the rumen simulation technique (RUSITEC). *J. Anim. Physiol. Anim. Nutr.* 97 1161–1169. 10.1111/jpn.12025 23278844

[B64] Zhi P. L. Han L. L. Li G. Y. Bao K. Kai Y. W. Chao X. (2013). Molecular diversity of rumen bacterial communities from tannin-rich and fiber-rich forage fed domestic Sika deer (*Cervus nippon*) in China. *BMC Microbiol.* 13:151. 10.1186/1471-2180-13-151 23834656 PMC3723558

[B65] Zhu Y. Wang C. Li F. (2015). Impact of dietary fiber/starch ratio in shaping caecal microbiota in rabbits. *Can. J. Microbiol.* 61 771–784. 10.1139/cjm-2015-0201 26361938

[B66] Zilliox C. Debeire P. (1998). Hydrolysis of wheat straw by a thermostable endoxylanase: adsorption and kinetic studies. *Enzyme Microb. Technol.* 22 58–63. 10.1016/S0141-0229(97)00105-1

