# Supplementary material for: Expression of a Recombinant Lentinula edodes Xylanase by Pichia pastoris and Its Effects on Ruminal Fermentation and Microbial Community in in vitro Incubation of Agricultural Straws
Source: Front Microbiol. 2018 Nov 30;9:2944. doi: 10.3389/fmicb.2018.02944 (PMC6283887; doi:10.3389/fmicb.2018.02944)
Supplement: Supplementary file 1 [file Image_1.PDF]

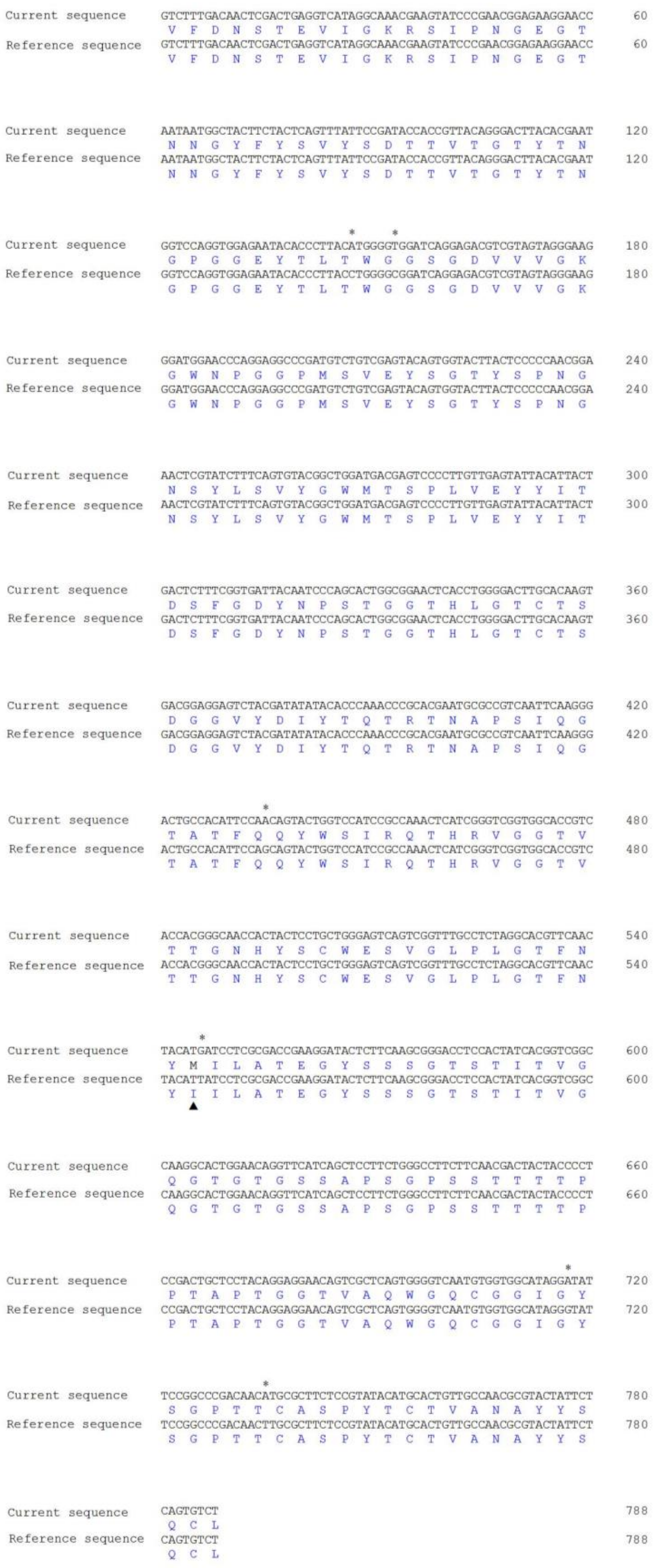

Figure S1 Alignment of gene sequence and amino-acid sequence for rLeXyn11A (GenBank no. MH349096) and reference sequence (GenBank no. AF411252.1). Asterisks indicate the differences of the gene sequences, and triangle indicates the differences of the amino-acid sequences.
